# Clinical challenges of biomechanical performance of narrow-diameter implants in maxillary posterior teeth in aging patients: A finite element analysis

**DOI:** 10.1371/journal.pone.0299816

**Published:** 2024-03-25

**Authors:** Saranyoo Prasitwuttisak, Nattapon Chantarapanich, Komsan Apinyauppatham, Kopchai Poomparnich, Samroeng Inglam

**Affiliations:** 1 Faculty of Dentistry, Thammasat University, Pathumthani, Thailand; 2 Department of Mechanical Engineering, Faculty of Engineering at Sriracha, Kasetsart University, Chonburi, Thailand; Yerevan State Medical University Named after Mkhitar Heratsi, ARMENIA

## Abstract

This study evaluated the biomechanical performance of narrow-diameter implant (NDI) treatment in atrophic maxillary posterior teeth in aging patients by finite element analysis. The upper left posterior bone segment with first and second premolar teeth missing obtained from a patient’s cone beam computed tomography data was simulated with cortical bone thicknesses of 0.5 and 1.0 mm. Three model groups were analyzed. The Regimen group had NDIs of 3.3 × 10 mm in length with non-splinted crowns. Experimental-1 group had NDIs of 3.0 × 10 mm in length with non-splinted crowns and Experimental-2 group had NDIs of 3.0 × 10 mm in length with splinted crowns. The applied load was 56.9 N in three directions: axial (along the implant axis), oblique at 30° (30° to the bucco-palatal plane compared to the vertical axis of the tooth), and lateral load at 90° (90° in the bucco-palatal plane compared to the vertical axis of the tooth). The results of the von Mises stress on the implant fixture, the elastic strain, and principal value of stress on the crestal marginal bone were analyzed. The axial load direction was comparable in the von Mises stress values in all groups, which indicated it was not necessary to use splinted crowns. The elastic strain values in the axial and oblique directions were within the limits of Frost’s mechanostat theory. The principal value of stress in all groups were under the threshold of the compressive stress and tensile strength of cortical bone. In the oblique and lateral directions, the splinted crown showed better results for both the von Mises stress, elastic strain, and principal value of stress than the non-splinted crown. In conclusion, category 2 NDIs can be used in the upper premolar region of aging patients in the case of insufficient bone for category 3 NDI restorations.

## 1. Introduction

Nowadays, the population of aging adults with more than 21 functional teeth throughout life has been increasing and is one of the important factors in the health of the elderly [1]. The demand for dental implants has increased and has become a promising treatment method to restore missing teeth [2] with high success rates and excellent predictability [3–6]. Nonetheless, aging patients frequently require special considerations in dental implant placement, which include medical problems, longer recovery time, and the reluctance to have invasive surgery. Therefore, minimally invasive surgery techniques for the elderly must be considered [7].

Standard-diameter implants (SDIs) of more than 3.75 mm in diameter are frequently used with highly successful long-term outcomes [6, 8, 9]. However, one of the challenging limitations for dental implant placement is alveolar crest atrophy with reduced bone width and height. Additional surgical treatment, such as maxillary sinus floor augmentation or lateral and/or vertical alveolar ridge augmentation, may be required to build up inadequate bone dimensions [8]; however, the risk of complications is potentially increased [10, 11]. In order to perform minimally invasive implant surgery and avoid ridge augmentation, a narrow-diameter implant (NDI) of ≤ 3.5 mm in diameter is one option that has a survival rate similar to SDIs. Furthermore, NDIs have clinical advantages in situations of limited restorative space, and NDIs can reduce the need for bone augmentation [6–9, 12].

NDIs in category 3 (diameter 3.3 to 3.5 mm) have strong evidence for use in all indications including the posterior area. Smaller implants in category 2 (diameter 2.5 to < 3.3 mm) are recommended for single tooth rehabilitation in areas of non-loading restoration [8]. According to several studies using finite element analysis (FEA), a smaller diameter implant presents higher levels of stress and strain at the implant crestal marginal bone level than conventional diameters, which anticipates a higher risk of bone loss [8, 13]. Therefore, NDIs have been employed primarily in specific conditions, such as implant placement at the incisal area, and are infrequently used in areas with high load of biting force such the molar teeth area [14]. However, many studies showed that age and gender are important factors that influence the maximum bite force. Occlusal force increases with age and growth and then declines in the elderly after 50 years of age due to decreased muscle mass and functional capacity [15]. On the other hand, the splinted prosthesis is an option to reduce stress on an implant and surrounding bone support. The splinting (or stabilization) is a binding restoration or implant that can improve the stability of the structure, enhance the overall surface receiving the load, and provide a better distribution of non-axial forces. According to several authors, splinting shows biomechanical benefits and promotes a load-sharing effect [16, 17] and lowers the tensile stress on the posterior area of short fixed bridges [17].

Several studies have used the FEA method to analyze and predict the biomechanical performance of stress induced at peri-implant bone and dental implant components [18–20]. A recent study [21, 22] of NDIs that supported single and splinted crowns reported that both have a high degree of patient satisfaction with peri-implant conditions and comparable peri-implant bone levels. Furthermore, they can be used successfully as a minimally invasive alternative to horizontal bone augmentation in the posterior region. Nevertheless, FEA studies of biomechanical performance on NDIs supporting non-splinted crowns and splinted crowns in the maxillary posterior region in aging patients are still lacking. Details of complications related to restorations, implant diameters, and aging patients of this therapeutic modality are still unknown. A few studies evaluated the biomechanics behavior of NDIs with splinted crowns with different thicknesses of cortical bone in the atrophic posterior maxilla region of aged patients [12, 20].

While the success of category 3 NDIs for load bearing is doubtless, nevertheless, debate still exists on the proper minimally invasive approach in implant planning for aging patients with reduced maximum bite force as well as the selection of an optimal implant diameter. The hypothesis is that category 2 NDIs can also effectively manage the atrophic posterior maxilla premolar area where the bone area is limited and also reduce the invasiveness in aging patients. Therefore, this study aimed to evaluate the biomechanical behavior using FEA of category 2 NDIs in the atrophic posterior maxilla premolar area with splinted and non-splinted crowns in elderly patients who have reduced maximum bite force.

## 2. Material and methods

The Ethical Review Sub-Committee Board for Human Research Involving Sciences at Thammasat University approved the research exemption of the present study, which followed the ethical principles of the Declaration of Helsinki, The Belmont Report, CIOMS guidelines, and the International Conference on Harmonization—Good Clinical Practice (ICH-GCP) guidelines.

In the process of data collection, in connection with authorized radiology personnel at Thammasat University, collecting patient information began on 24 June 2022 and the required radiographs were received on 30 June 2022.

After the authorization, the researcher obtained the required radiograph DICOM files from the personnel of the Radiology Department at Thammasat University that was authorized to provide patient information. The only information obtained was the radiograph without any information that could be traced back to the patient. During and after the process of obtaining the information, all authors could not access the patient’s information and did not require any other information other than the obtained radiograph.

### 2.1 Bone models and implant designs

Based on Schiegnitz and Al-Nawas [6] for this study, a category 2 narrow-diameter two-piece implant (diameter 3.0 mm) and a category 3 narrow-diameter two-piece implant (diameter 3.3 mm) were used in three groups. The Regimen (R) group received two-piece implants of category 3 narrow diameter of 3.3 mm restored with non-splinted crowns. The Experimental-1 (E-1) group received two-piece implants of category 2 narrow diameter of 3.0 mm restored with non-splinted crowns, and Experimental-2 (E-2) group received two-piece implants of category 2 narrow diameter of 3.0 mm restored with splinted crowns (Table 1). The two-piece implant with the smaller diameter restored with splinted crowns was used as a comparison parameter. The clinical practice guidelines produced from the work of Schiegnitz and Al-Nawas, 2018 [6] suggested choosing an implant-supported single crown for NDI category 3 (R group). All implant models were 10.0 mm in length.

**Table 1:** Experimental design groups.

Group	Model	Implant diameter (mm)	Cortical bone thickness (mm)	Splinted/non-splinted crown
Regimen	R-05	3.3	0.5	Non-splinted crown
	R-10		1.0	
Experimental 1	E1-05	3.0	0.5	Non-splinted crown
	E1-10		1.0	
Experimental 2	E2-05	3.0	0.5	Splinted crown
	E2-10		1.0	

R-05 = Regimen group with 0.5 mm cortical thickness

R-10 = Regimen group with 1.0 mm cortical thickness

E1-05 = Experimental group-1 with 0.5 mm cortical thickness

E1-10 = Experimental group-1 with 1.0 mm cortical thickness

E2-05 = Experimental group-2 with 0.5 mm cortical thickness

E2-10 = Experimental group-2 with 1.0 mm cortical thickness

**Fig 1:**
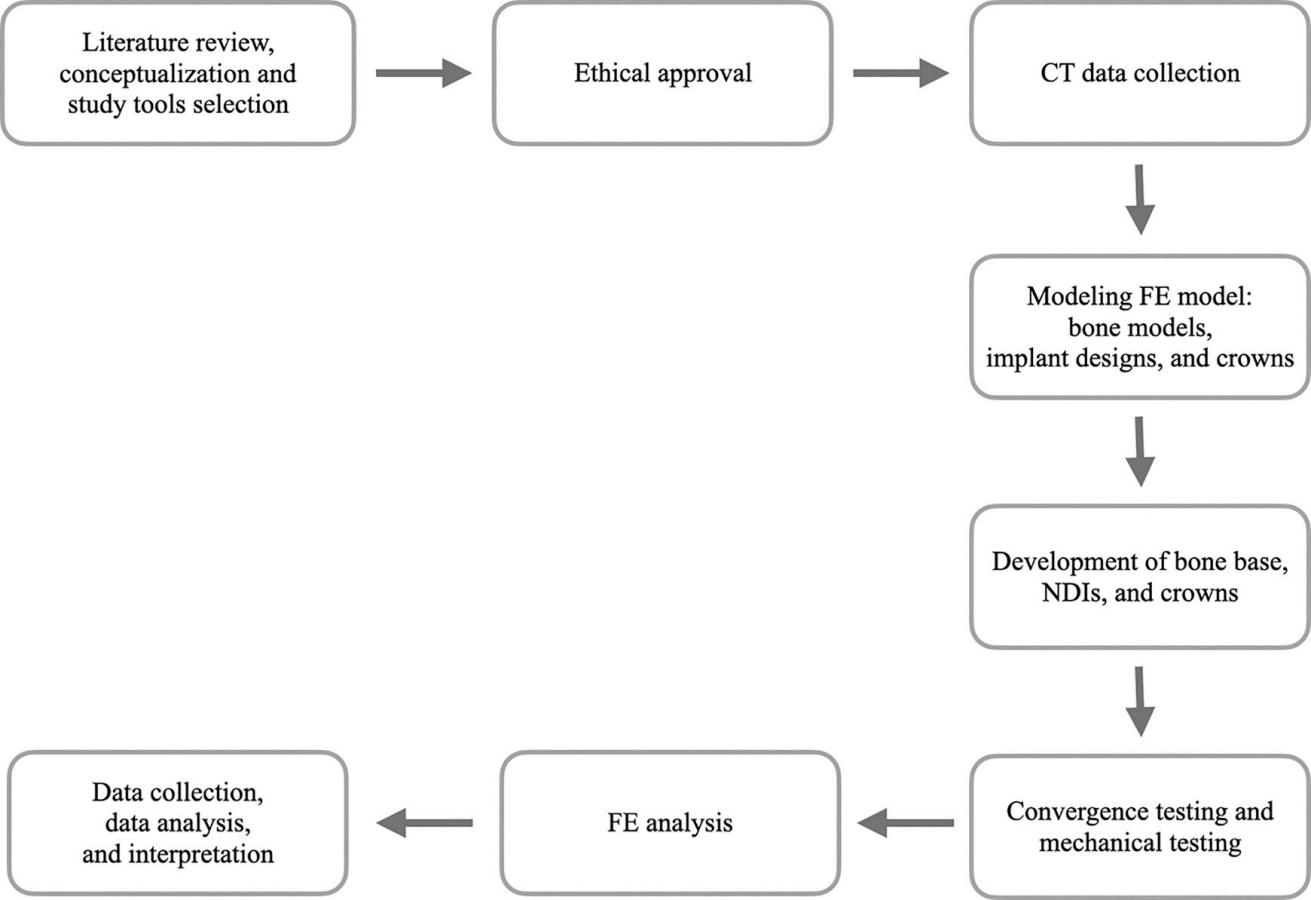
Workflow of the study.

**Fig 2:**
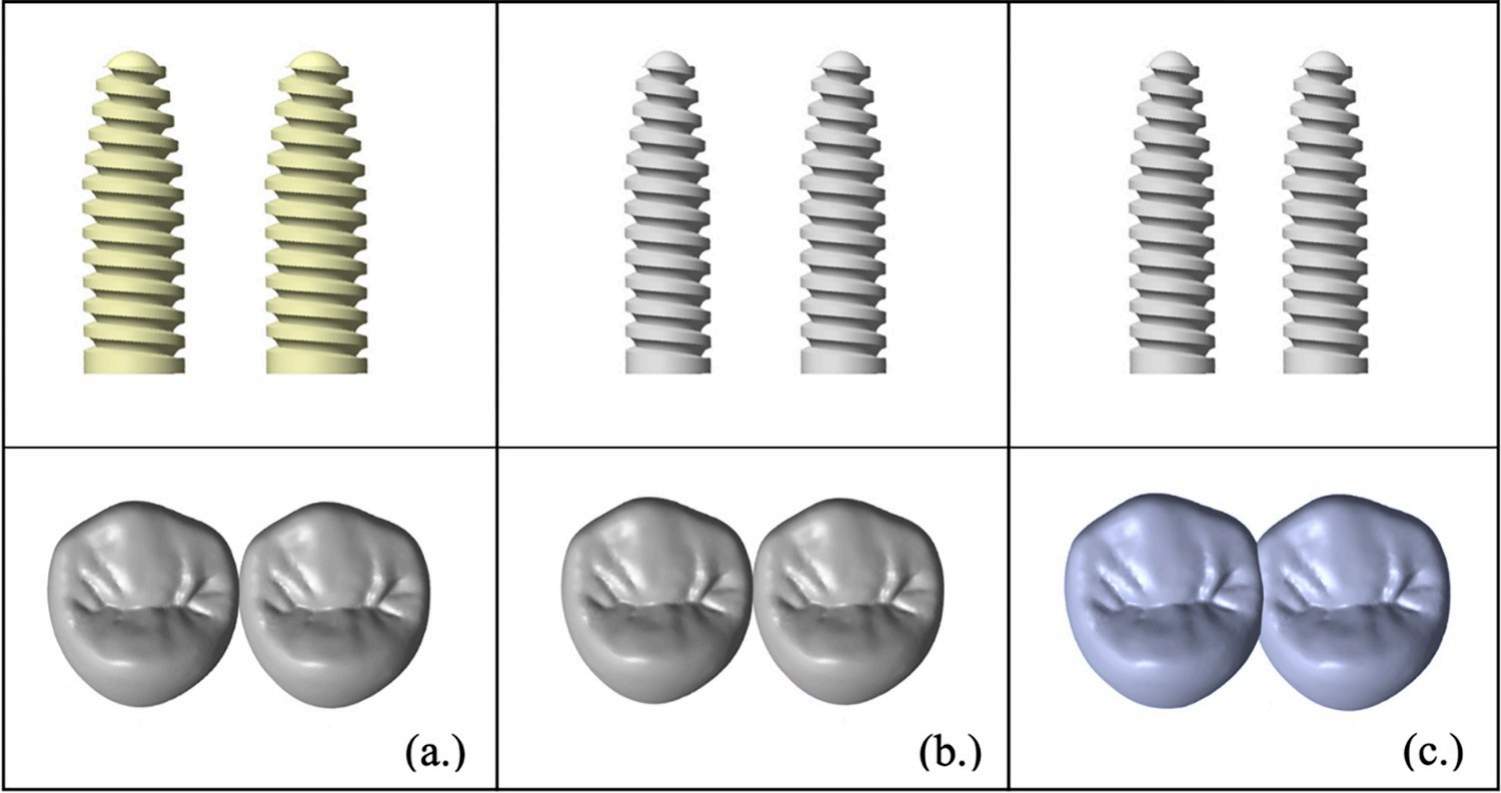
CAD model of splinted and non-splinted narrow-diameter implants. **(a)** Regimen group, **(b)** Experimental-1 group, and **(c)** Experimental-2 group.

**Fig 3:**
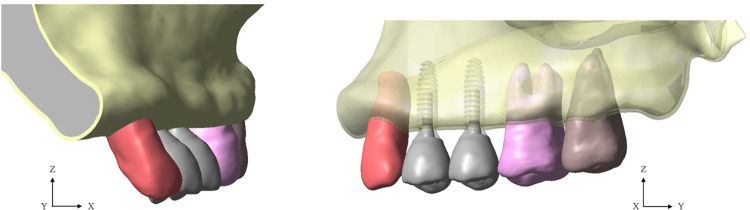
CAD models of narrow-diameter implants restored with splinted and non-splinted crowns in posterior atrophic maxilla.

The NDI implant components (implant, abutment, screws) used in this study were modelled and simulated according to the configuration of the Straumann bone level tapered (BLT) implant (Institut Straumann AG, Basel, Switzerland). The Straumann BLT implant fixture (length = 10 mm, external diameter = 3.3 mm [Narrow CrossFit^®^] for R group, and external diameter = 2.9 mm [Small CrossFit^®^] for E1 and E2 group) is fitted with a 15° conical-cylindrical CrossFit^®^ internal connection with four internal grooves. The crown is connected to a screw-retained abutment (abutment gingival height = 1 mm, abutment height = 5.5 mm, straight standard abutment). Fig 1 illustrates the workflow of the present study. Fig 2 illustrates the sets of implants and restorations in each group. Fig 3 shows the positions of the implants and the posterior maxillary bone model in 0.5 mm and 1.0 mm cortical thicknesses.

The maxilla model used in this study was obtained from of an upper left posterior bone segment belonging to a 74-year-old edentulous female with bone dimensions adequate for the placement of an implant length of 10 mm at the area of the first and second premolar teeth. The DICOM file containing the CT data of the patient was imported into the 3D Slicer Software image processing program (http://www.slicer.org) to construct a 3D model of the maxilla region which included cortical bone, cancellous bone, and teeth. Different thresholds were applied to different regions of the CT images to separate cortical bone and teeth from other parts. The cortical bone and teeth were recorded in the form of stereolithography (.stl) file format. The surface of both models was later improved to fix incomplete topology as well as finalize their shapes in computer-aided design software (VISI, Hexagon AB, Sweden). The model region of interest included the arch ranging approximately from the canine to the second molar. In order to simulate the condition under interest of this study, both pre-molars were removed. The canine and molars were in placed with the maxilla. The maxilla model was subtracted from these teeth which produce the complete exterior surface of cortical bone. The cancellous bone was internally offset from the obtained external surface of cortical bone by 2 mm. The external cortical bone layer previously described was then subtracted to the cancellous bone layer to produce the complete cortical bone of maxilla. The cortical bone design was reconstructed with thicknesses of 0.5 mm and 1.0 mm [23], and simulation of the bone model density was completed in accordance with the previous studies [20, 24]. Prosthetic crown models were created using the VISI software and standardized as upper left first and second premolar zirconia crowns. FEA analyzed the bone base, NDIs, and crowns with a commercial finite element software package (Marc Mentat, MSC Software Corp., USA) using a multifrontal sparse type solver. The mechanical properties of the structural models, which included the zirconia crown, abutment, titanium-zirconium (Ti-Zr) NDI, cortical bone, and cancellous bone, were defined by individual values of modulus of elasticity and Poisson’s ratio (Table 2). All model materials were isotropic and linearly elastic. A mesh refinement was completely reconstructed in the regions of interest for this study. The element type employed in this study was the 4-node tetrahedral element. A convergence study was conducted by generating different element sizes. It was found that an element length <0.70 mm provided insignificant changes in bone stress.

**Table 2:** Mechanical properties of the materials used in the study.

Materials	Young’s modulus (MPa)	Poisson ratio	Reference
Zirconia crown	200,000	0.31	Lee et al., 2021 [25]
Abutment (Ti)	110,000	0.30	Grandin et al., 2012 [19] Cinel et al., 2018 [20]
Screw (Ti)	110,000	0.30	Grandin et al., 2012 [19] Cinel et al., 2018 [20]
Ti-Zr implant	100,000	0.30	Cinel et al., 2018 [20] Pérez RA et al., 2020 [26]
Cancellous bone	1,370	0.31	Cinel et al., 2018 [20] Geng J, 2001 [24]
Cortical bone	13,700	0.30	Cinel et al., 2018 [20]

**Fig 4:**
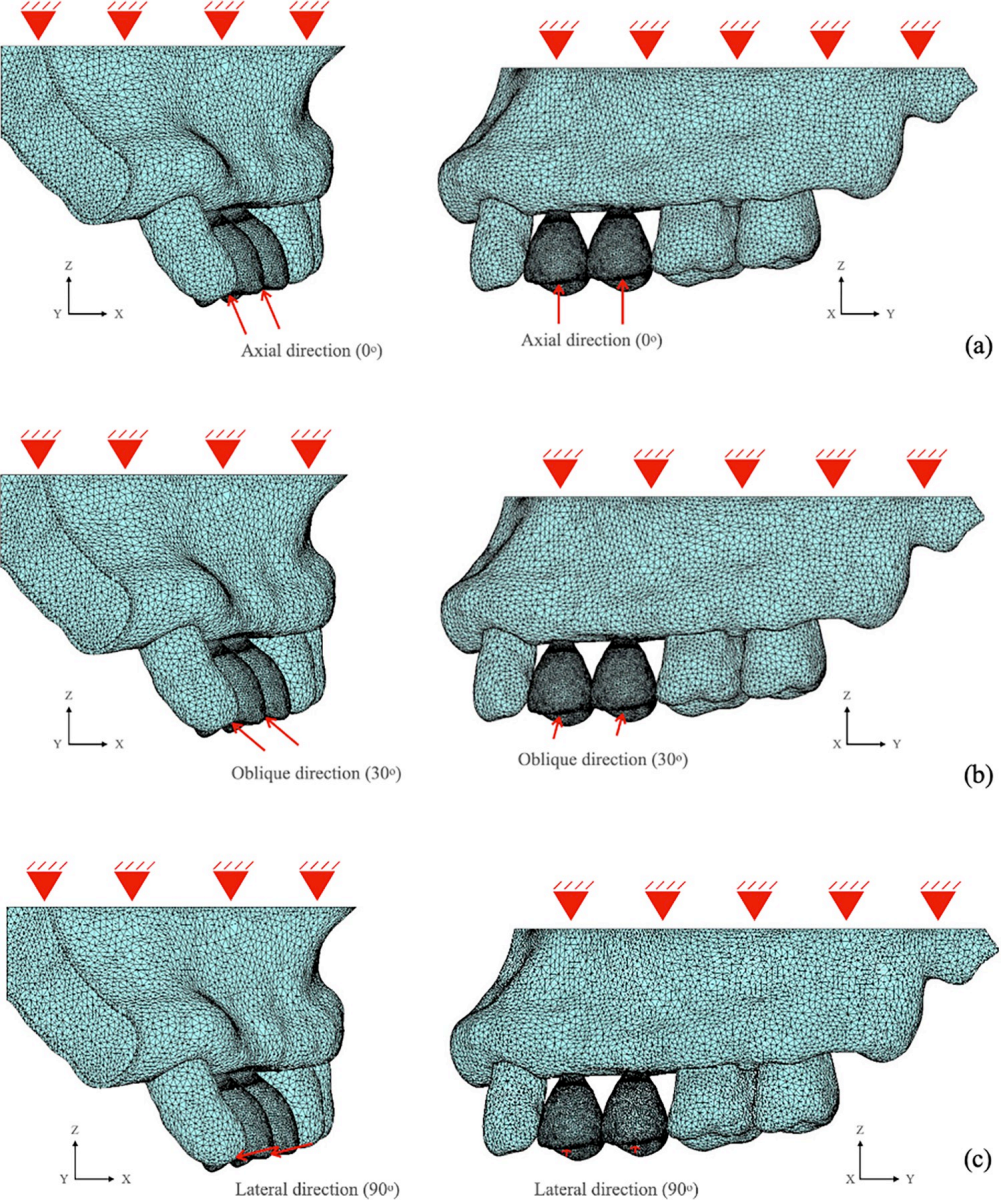
Mesh element models of splinted and non-splinted narrow-diameter implants with boundary conditions and loading application points.

The total number of elements in the models ranged from 424,138 to 436,488, and the total number of nodes ranged from 103,018 to 105,876 (Table 3). The element model with boundary conditions and loading application points are indicated in Fig 4.

**Table 3:** Total number of nodes and elements for each model.

Group	Model	Element	Node
Regimen	R-05	428,773	105,265
	R-10	436,488	105,876
Experimental-1	E-05	424,138	103,974
	E-10	429,670	103,980
Experimental-2	E-05	425,375	104,111
	E-10	425,935	103,018

From the simulation limits, contacts of the fundamental structural models were set as bonded contact between the crown and abutment, between cancellous bone and cortical bone, and between the implant and bone. The bonded contact simulates the clinical conditions following successful implant treatment, bone-implant osseointegration and the fixing of all implant components as a single unit. In all cases under consideration, the conventional loading protocol (loading condition after full osseointegration loading), which was assumed to be 56.9 N to represents the bite force of a single premolar tooth in healthy adults, was applied to the crown surface and is equivalent to the bite force of the anterior teeth [27]. The load was applied in three directions: axial load (along the implant axis), oblique load at 30° (30° to bucco-palatal plane compared to the tooth vertical axis), and lateral load at 90° (90° in bucco-palatal plane compared to the tooth vertical axis).

The primary outcomes of the main structures of interest recorded in this study were the NDI and peri-implant crestal bone (cortical bone). The prosthetic crown, abutment, and abutment screw were created to simulate only the clinical situation; therefore, the outcomes of these simulations were not included. All implant models underwent von Mises stress while peri-implant bone models underwent elastic strain analysis [28], minimum principal values of stress (Pmin), and maximum principal values of stress (Pmax) [12, 13, 20, 29, 30]. In accordance with similar studies [28, 31], the present research tested the risk of overloading at different categories of NDIs and at the crestal bone for the different prosthetic solutions. The results of von Mises stress on the implants were considered according to the yield strength properties of a NDI Ti-Zr alloy 689 MPa [26]. The results of the peri-implant crestal bone response in elastic strain were evaluated by Frost’s mechanostat theory [32]. Additionally, the bone response in Pmax or Pmin were evaluated by the compressive strength [12, 13]. The cortical bone strength was assumed to withstand compressions that ranged from 170 to 190 MPa and 100 to 130 MPa under tension [20]. Stress values that exceed these thresholds might lead to deformation of the bone structure [20, 33].

### 2.2 Mechanical testing

The FEA method approximates a mechanical issue through numerical analysis, which has less precision than a result obtained from mechanical testing. Therefore, it is necessary to validate the FE result using physical experiment to confirm the feasibility of the FE model [34]. The E2-10 model configuration was selected for mechanical testing to validate the model. The maxillary bone model was 3D-printed from polylactic acid filament (Verbatim, Mitsubishi Kagaku Media Co., Ltd., Japan) using the Zortrax M300 Plus fused deposition modeling machine (Zortrax S.A., Poland).

**Fig 5:**
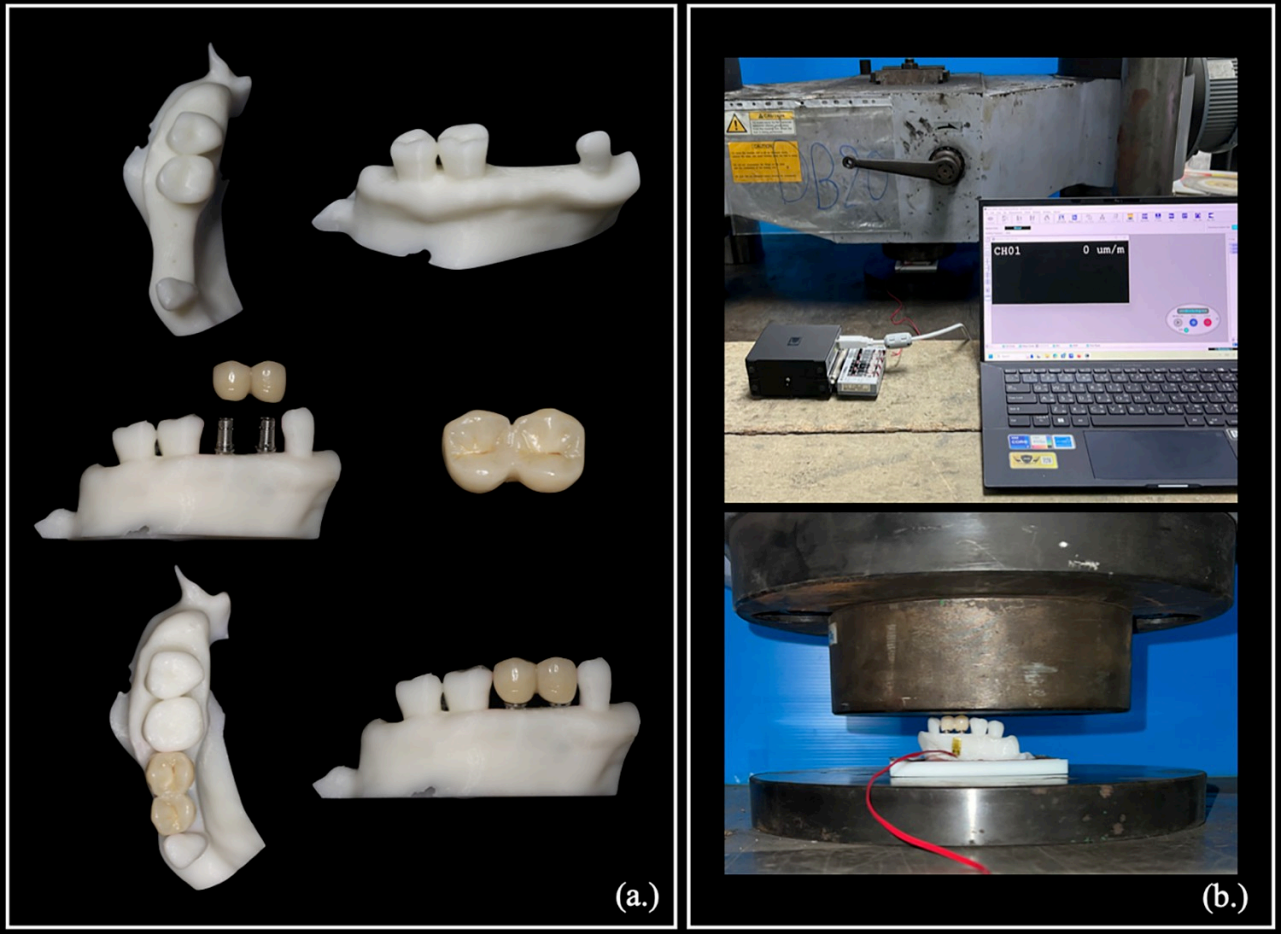
Experimental group-2 with a 1.0 mm cortical thickness model used for validation of the mechanical test. **(a.)** Maxilla bone model and narrow diameter implant restored with splinted-crown and **(b.)** mechanical test setup.

The printing parameters included 0.2 mm layer thickness, 210°C nozzle temperature, 60°C platform temperature, 30 mm/s printing speed, 45° cross-hatch raster angle, and a rectilinear infilled pattern. The 3D-printed bone model had mechanical properties corresponding to the study of Chaitat [35]. Two category 2 NDI fixtures were placed in the edentulous site and restored with splinted zirconia crowns simulated as in the FE model. A 100-N mechanical load generated by a universal testing machine (UH-1000, Shimadzu Corp., Japan) was applied in the axial direction to the superstructure of the model. Elastic strain was measured by a strain gauge (KFP-2-120-C1-65L1M2R; Kyowa Electronic Instruments Co., Ltd., Japan) placed on the outer palatal surface of the bone structure (Fig 5). The data logger (EDX-10B, EDX-11A, Kyowa Electronic Instruments Co., Ltd., Japan) was used to capture the measured strain values. The measured values and the FE results obtained using the same geometric model were then compared.

## 3. Results

### 3.1 von Mises stress in axial load

**Fig 6:**
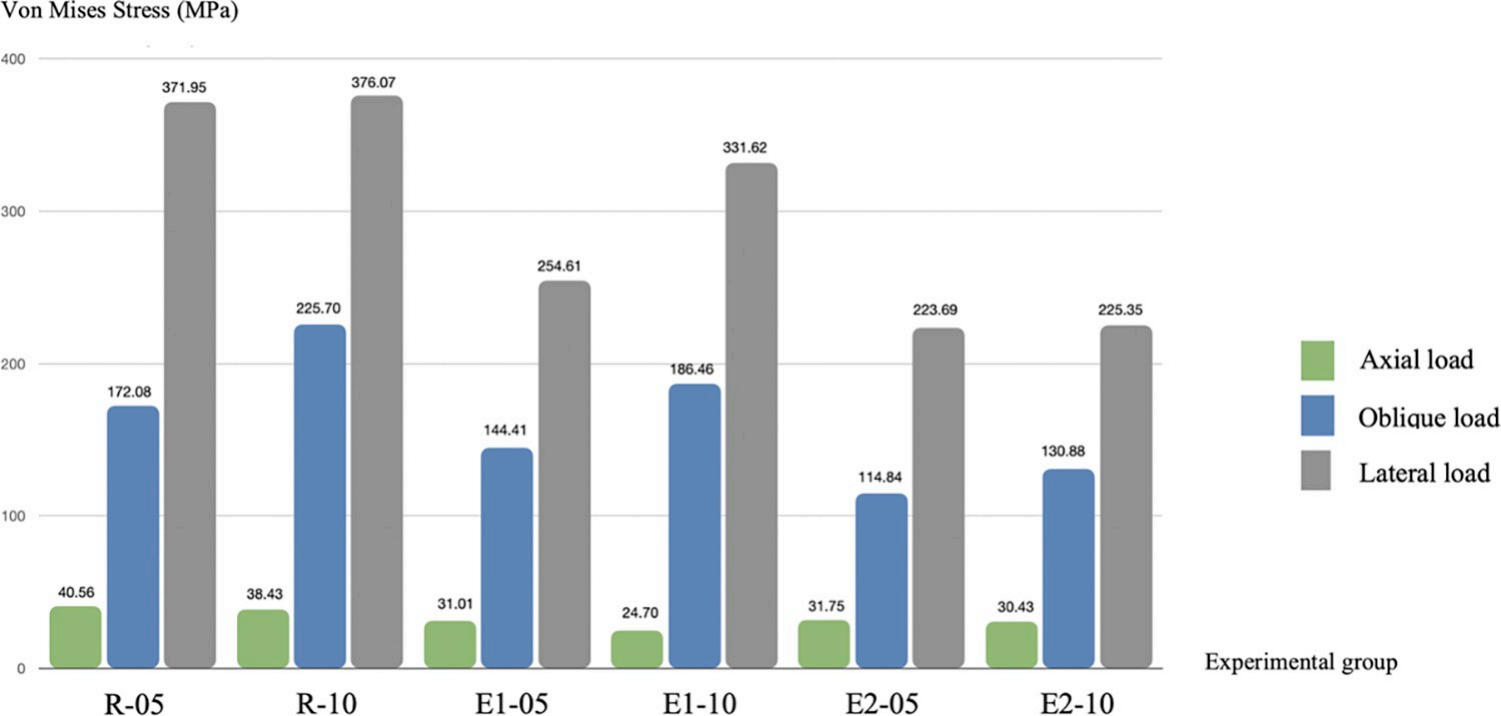
Results of the von Mises stress values in narrow-diameter implants in all groups. Cortical thickness: -05 = 0.5 mm and -10 = 1.0 mm.

**Fig 7:**
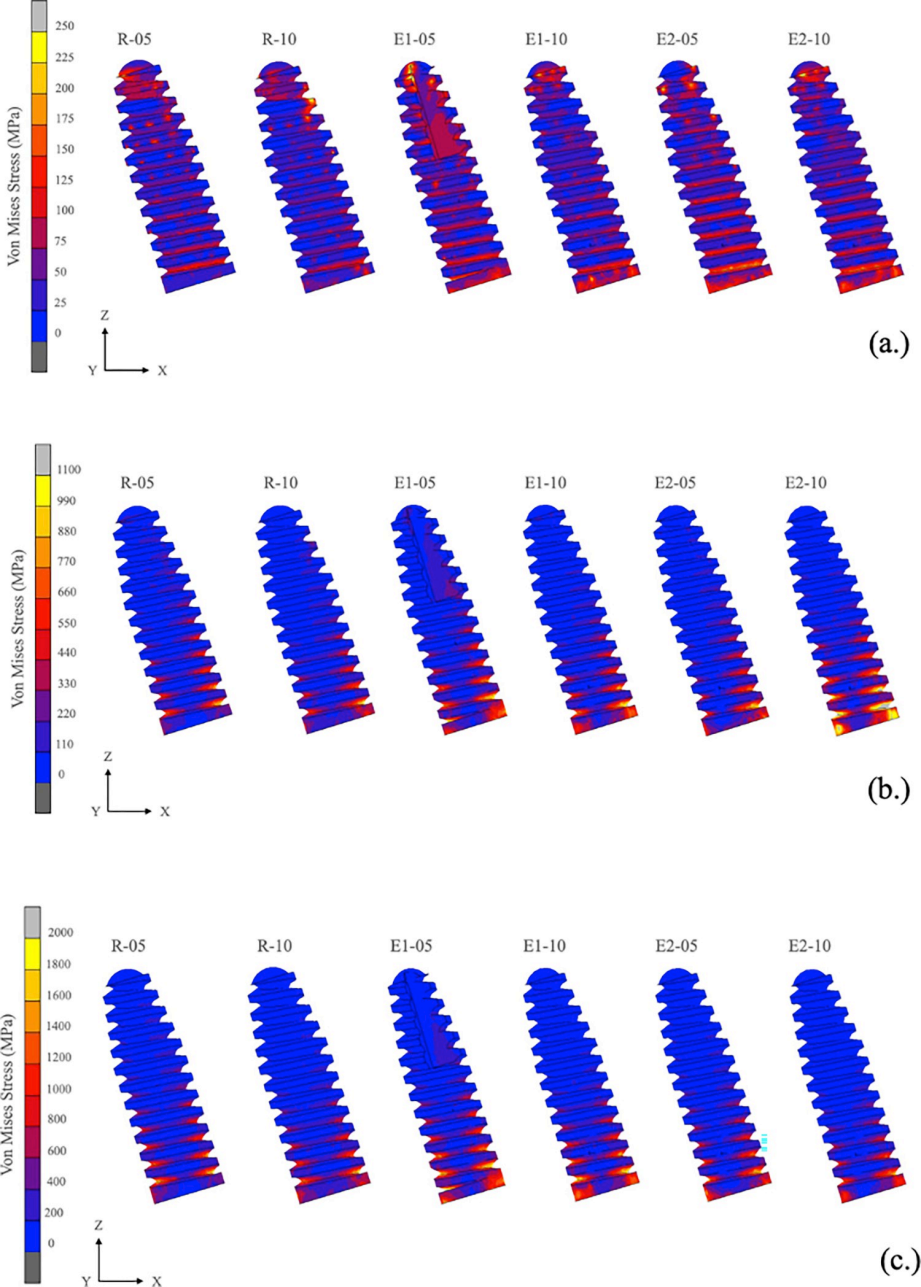
Distribution patterns of von Mises stress in narrow-diameter implants. **(a.)** Axial load direction, **(b.)** oblique load direction, and **(c.)** lateral load direction. Cortical thickness: -05 = 0.5 mm and -10 = 1.0 mm.

Maximum von Mises stress values were observed around the platform of the implant fixture and abutment connection, which corresponded to the difference in the implant tooth position and volume of surrounding bone tissue. Fig 6 shows the results of von Mises stress in each group. The highest value of von Mises stress was observed in the R group (40.56 MPa), followed by the E-2 group (31.75.00 MPa) and the E-1 group (31.01 MPa). Fig 7 shows the distribution patterns generated by von Mises stress after applying the difference of 3 direction loads in NDIs.

### 3.2 von Mises stress in oblique load

In the oblique load and lateral load directions, increased von Mises stress values were observed in all groups compared to the axial direction. The highest von Mises stress value in the oblique load direction was observed in the implant of the R group (225.70 MPa), followed by the E-1 group (186.46 MPa), and the lowest von Mises stress value was observed in the E-2 splinted-crown group (130.88 MPa).

### 3.3 von Mises stress in lateral load

The lateral load direction represents the parafunctional habits similar to bruxism or tooth clenching. The highest von Mises stress value of the lateral load was observed in the implant of the R group (376.07 MPa) followed by the E-1 group (331.62 MPa) and E-2 group (225.35 MPa).

### 3.4 Elastic strain results of peri-implant bone

**Fig 8:**
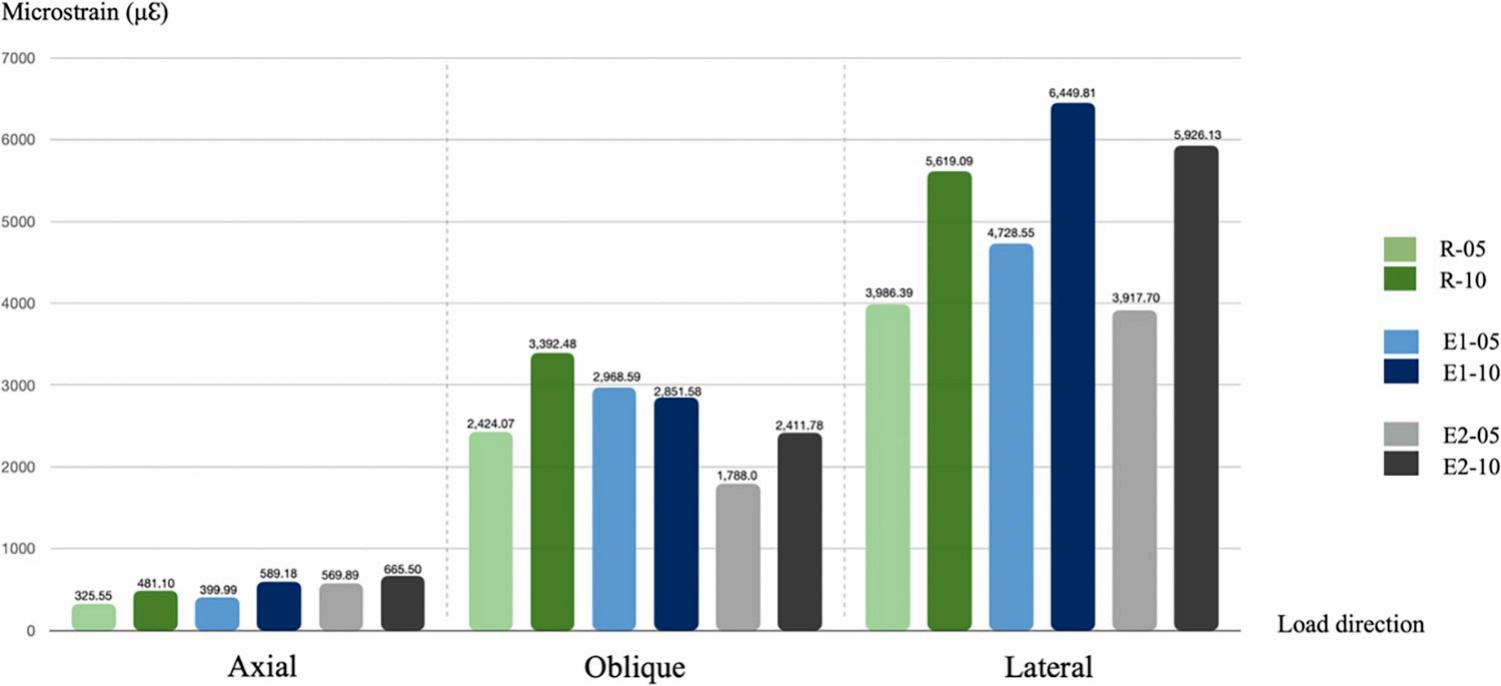
Results of elastic strain on the surrounding bone in all groups. Cortical thickness: -05 = 0.5 mm and -10 = 1.0 mm.

**Fig 9:**
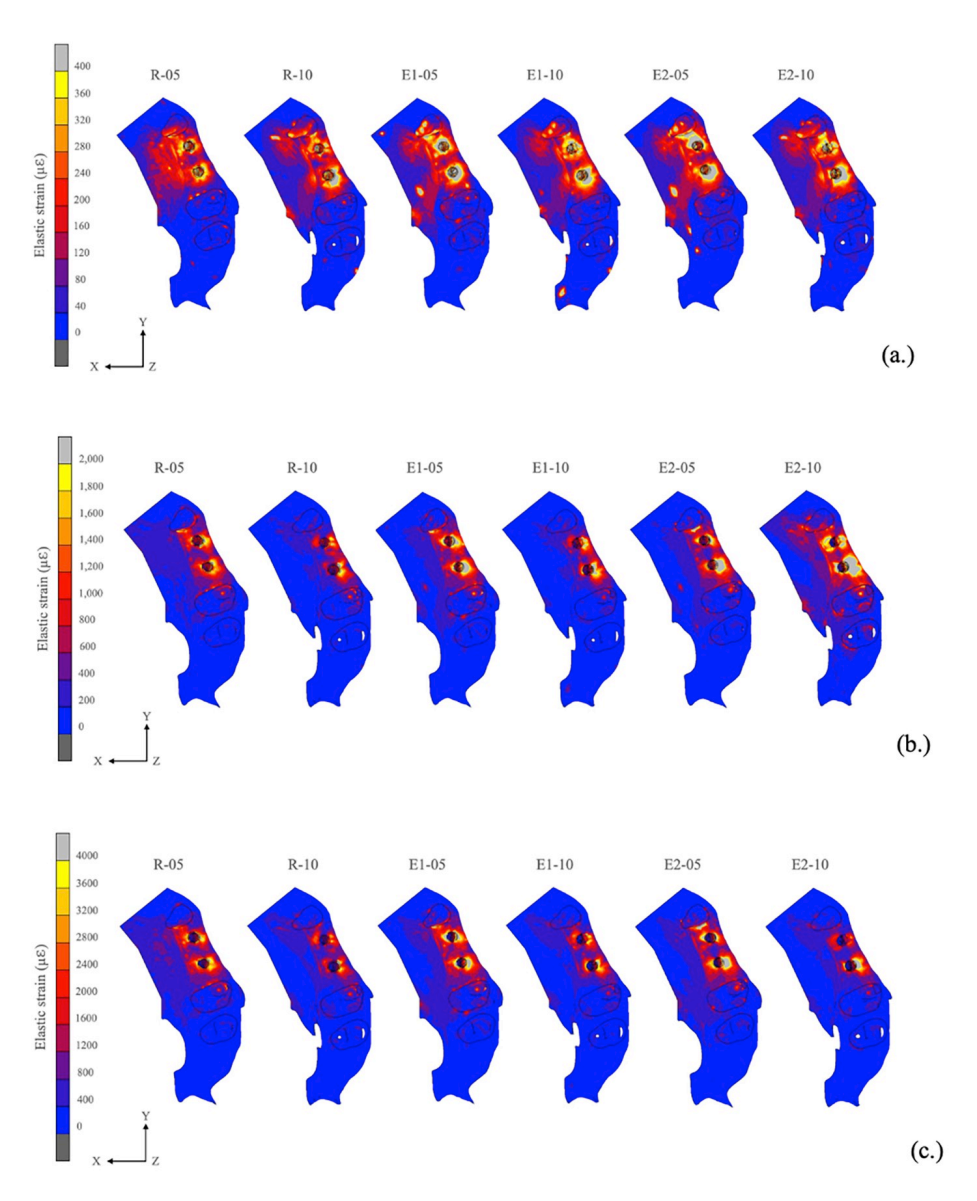
Distribution pattern of elastic strain on surrounding bone. **(a.)** Axial load direction, **(b.)** oblique load direction, and **(c.)** lateral load direction. Cortical thickness: -05 = 0.5 mm and -10 = 1.0 mm.

Fig 8 shows the results of elastic strain in all groups. The values of peri-implant bone elastic strain were analyzed using Frost’s mechanostat theory. The results of elastic strain in the axial load direction ranged from 325.55 to 690.77 microstrain (μℇ), which were comparable in all groups. The elastic strain results for the oblique load ranged from 1,788 to 4,043.58 μℇ. In the lateral load direction, the peri-implant bone elastic strain value ranged from 3,917.70 to 7,051.27 μℇ. Fig 9 shows the distribution pattern generated by elastic strain in the peri-implant crestal marginal bone after application of the difference of the three-directional loads in the NDIs.

### 3.5 Principal value of stress results of peri-implant bone

**Fig 10:**
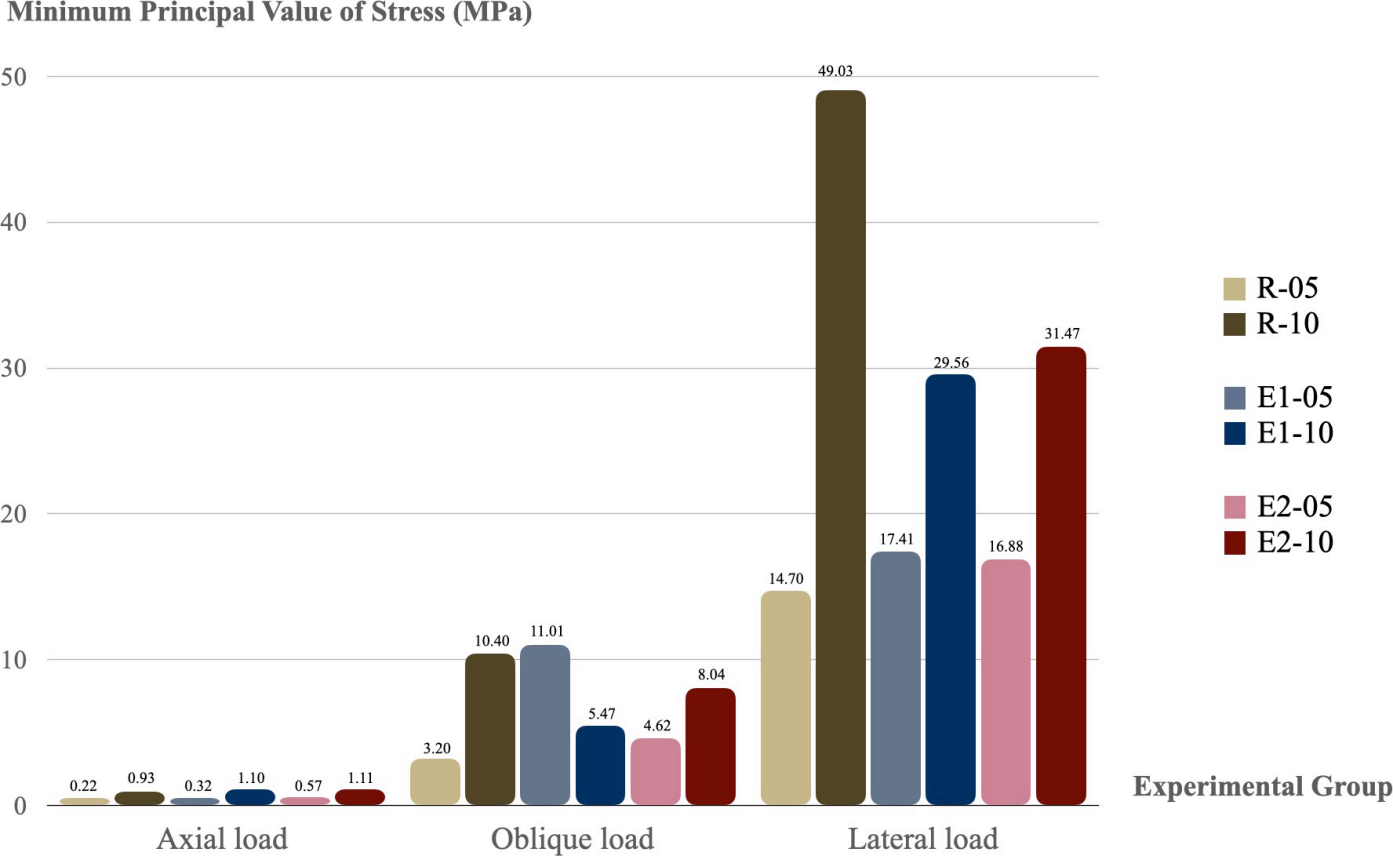
Minimum principal value of stress on the surrounding bone in all groups. Cortical thickness: -05 = 0.5 mm and -10 = 1.0 mm.

**Fig 11:**
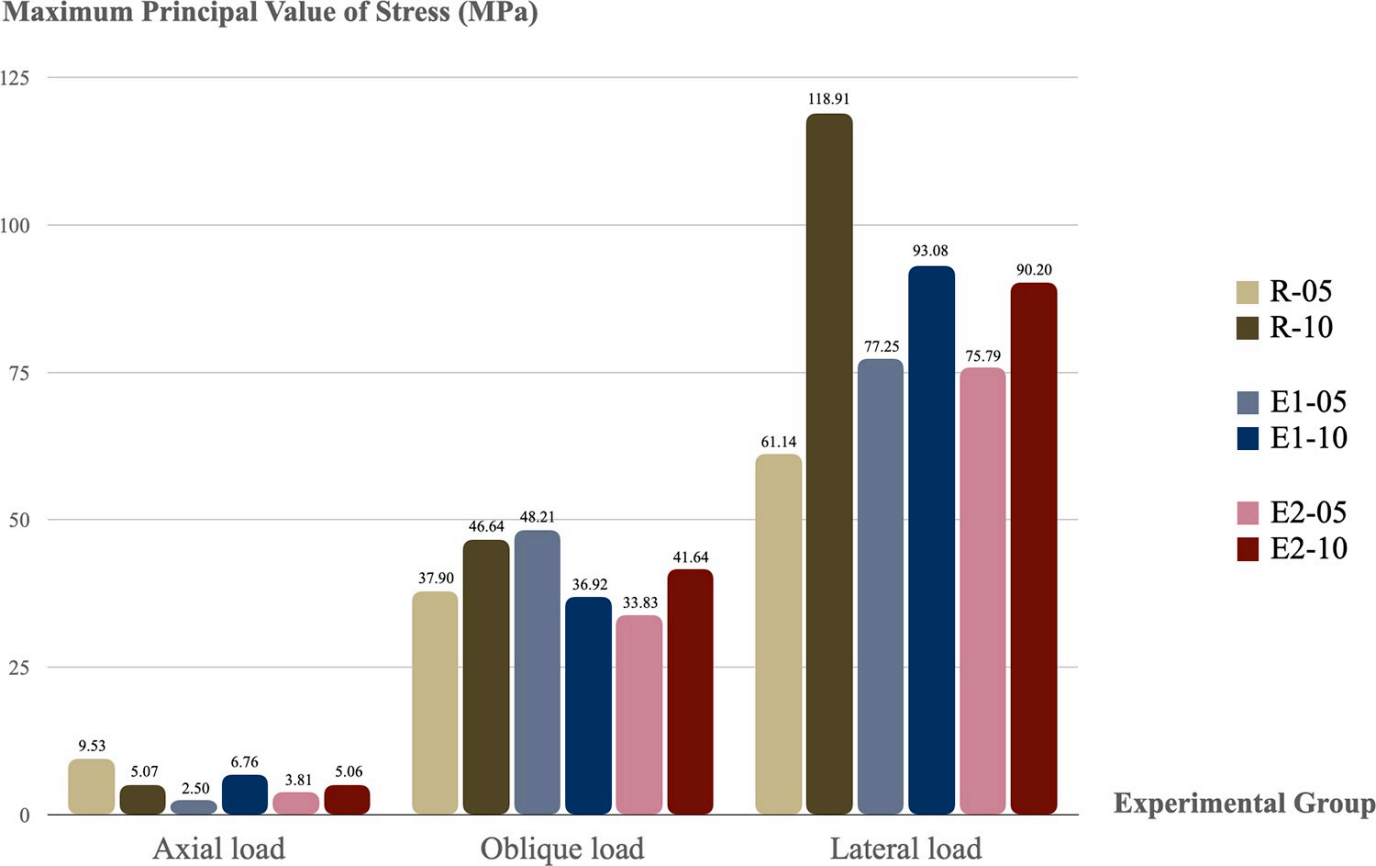
Maximum principal value of stress on the surrounding bone in all groups. Cortical thickness: -05 = 0.5 mm and -10 = 1.0 mm.

In all models, both Pmin and Pmax results were lateral load > oblique load > axial load. Peak Pmin and Pmax values were observed in the lateral loading direction in the R-10 group (49.03 MPa and 118.91 MPa, respectively). The results of Pmin are shown in Fig 10 and the results of Pmax are shown in Fig 11.

### 3.6 Mechanical validation

While the strain measured by FEA was 65.7 μℇ, the average strain from mechanical testing was 60.0 μℇ. The estimated 9.5% difference between the FE result and the mechanical testing was observed to be sufficient to prove that the numeric conditions used in the FEA were appropriate.

## 4. Discussion

Currently the aging population is a global phenomenon [7]. One of the main problems of aging people is tooth loss [1]; however, dental implant rehabilitation may improve oral health‐related quality of life [6, 8]. A greater number of elderly persons with general health issues require special considerations when receiving dental implant treatment [7]. After tooth extraction, it is inevitable the alveolar crest will atrophy and reduce in width and height [20], which creates a challenging limitation in the treatment of dental implants [6]. In this situation, additional surgical procedures may be necessary to augment the insufficient bone volume and repair the vertical, horizontal, or sagittal interocclusal relationships [8]. The augmentation procedure is time and cost consuming along with a higher risk of complications [6, 8]. Furthermore, minimally invasive procedures should be considered when giving implant treatment in elderly adults [7]. A more conservative approach is the NDI, which was created to avoid these additional procedures and facilitate successful restoration [20].

In biomechanical aspects, it was reported that the macrodesign of dental implants, including fixture length and diameter, influences implant predictability and success [36]. However, the influence of dental implant diameter on long-term survival outcome is secondary. The important factors that affect long-term survival of dental implants in the atrophic maxilla posterior area include proper surgical technique, achievement of adequate implant primary stability, and proper pre- and post-operative oral hygiene maintenance [37].

The force effects on the surrounding tissue and implant material, as well as the generated distributed stress following loading on a bone-implant contact, are difficult to study in vivo [20]. As a consequence, stress analysis using FEA has been useful and most widely used to explain the biomechanical performance of implant restorations [12, 20]. The load sharing results of splinting prostheses is more favorable compared to non-splinted crowns [17, 38]. A splinted crown on an implant showed better biomechanical performance of maximum von Mises stress and lower cortical bone stress [17, 39]. Implant fracture is one of the main complications of NDIs [40]; therefore, the choice of implant material is an important factor [20]. An implant fixture made of Ti-Zr has superior mechanical properties compared to titanium (Ti). A Ti-Zr narrow-diameter implant material has a yield strength up to 689 MPa [26] and is 10–15% higher than Grade 4 Ti in tensile elongation and fracture toughness [41]. Similar to a previous study [20], this current study measured von Mises stress in three load directions. None of the stress results exceeded the yield strength of Ti-Zr implant material.

In this present study, the stress analysis evaluated the vertical and oblique loads, which simulated the complex chewing load in normal conditions, and the horizontal loading simulated bruxism habits. Interestingly, the results of the first premolar and second premolar von Mises stress analysis demonstrated that the axial load of the three groups had comparable maximum von Mises stress values as in a previous study [31]. A study by Al Aali et al, 2019 [21] reported that the plaque index, bleeding on probing, and probing depth did not significantly differ between splinted and non-splinted crowns. Peri-implant conditions and peri-implant bone levels were comparable. However, compared to other fixed partial restorations, single crown restorations provide a more comfortable approach by better emergence profiles and better access for oral hygiene maintenance [42]. Therefore, in order to avoid biological complications, it is not necessary to use a splinted crown to withstand the axial load. The maximum stress value in the oblique load direction observed in the R group was 17.39% higher than the E-1 group and 42.02% higher than the E-2 group. The maximum stress value in the lateral load direction observed in the R group was 11.82% higher than the E-1 group and 40.08% higher than the E-2 group. In the lateral load direction, all models exhibited the highest von Mises stress values.

The highest stress result in the lateral direction of the R-10 (376.07 MPa) was 55.3% of the static tensile limit of a Ti implant abutment and Ti screw (680 MPa). Likewise, the highest stress result was 54.5% of the yield strength of a Ti-Zr implant fixture (689 MPa). In clinical conditions, lateral jaw movement activity occurs in the bruxism habit, characterized by the clenching or grinding of teeth. Frequent sleep bruxism occurs in 13% of adults [43]. Our study showed lateral stresses in the implants reach 375 MPa values; however, a study by M. Janecek, 2015 reported that the fatigue life of titanium alloy at a stress amplitude <400 MPa can be tolerated up to 10^10^ cycles in tension-compression and rotating bending tests [44]. Therefore, the 375 MPa stress value is under a fatigue endurance of Ti-Zr dental implant.

The bone surrounding an implant is one of the importance factors that influences osseointegration [8, 45]. A study by Zarb [46] reported the posterior maxilla has the lowest bone density and also the thinnest cortical bone thickness. Ko et al. [23] reported that the posterior maxillary cortical bone thickness in the elderly was around 0.5 to 1 mm. The crestal cortical bone was thinner on average in the older group compared to the younger group, especially on the posterior maxilla where it was much thinner in the older group [45]. The bone model in this study was obtained and reconstructed from a patient’s cone beam computed tomography images to represent the character of the natural bone geometry. Naturally, in the alveolar ridge, the more posterior alveolar ridge is thicker than in the anterior in bucco-lingual width. Therefore, the results of the biomechanics performance demonstrated better elastic strain in the peri-implant bone of the second premolar tooth than the first premolar tooth. In the stress analysis, the elastic strain in the axial load direction in all groups was within the adapted window (physiologic window) of Frost’s mechanostat theory [32], while the results of the oblique load were within the mild overload window. The lateral load results were within the pathologic overload window. In this zone, bone resorption will occur. However, in a clinical scenario the lateral direction (90°) of occlusion is not common but occurs in tooth grinding and in patients with bruxism and tooth clenching habits [43].

The results of elastic strain in the axial load direction were comparable in stress distribution in all groups. However, in the oblique and lateral loads, the splinted crown group showed better results in elastic strain than the non-splinted crown group, and these results were smaller than the R group. Bone tissue is brittle, therefore, this study used Pmin and Pmax to evaluate the risk indicators for peri-implant bone resorption, as similarly reported in previous studies [12, 20, 29, 30]. The results of Pmin and Pmax values in all groups were under the threshold of the compressive stress and tensile strength of cortical bone. These results demonstrated that the peri-implant bone can withstand the loading applied, and peri-implant bone resorption did not occur. The results of Pmin and Pmax in the axial load direction were comparable in all groups. In addition, the highest principal stress values were noted in the Pmax of the lateral loading direction in the R-10 group. However, under oblique and lateral loads, the splinted-crown group performed with better results than the non-splinted crown group in Pmin and Pmax values, and these results were less than those of the R group. Many previous studies [12, 16, 17] demonstrated that the biomechanical advantages of the splinting prosthesis concept were unquestionable. In addition, it was proven that the peri-implant overload volume around NDIs with a splinted restorations was even less than around the non-splinted implants with a regular diameter, which was consistent with the Valera-Jiménez et al., 2020 study [12].

The one limitation of this study is the complexity of the oral structure that rarely transmits stress precisely as predicted. The results of this FE study involved a large number of aging patients or patients who needed minimally invasive surgery. It is important in the future to consider evaluating the effects and results of category 2 NDI restorations in the region of the posterior teeth.

## 5. Conclusion

From a biomechanics point of view, the results of this study showed that category 2 NDIs (2.5 to < 3.3 mm) can be used in the upper premolar region of aging patients in the case of insufficient bone for category 3 NDI (3.3 to 3.5 mm) restorations. Additionally, the splinted crown can be helpful in conditions of non-axial load for a better load sharing effect.

However, in clinical studies on oral rehabilitation using NDIs to support non-splinted and splinted crowns in aging patients is complex. Therefore, long-term follow-up is needed in aging patients.
